# Prophylactic Melatonin for Delirium in Intensive Care (Pro-MEDIC): study protocol for a randomised controlled trial

**DOI:** 10.1186/s13063-016-1751-0

**Published:** 2017-01-06

**Authors:** F. Eduardo Martinez, Matthew Anstey, Andrew Ford, Brigit Roberts, Miranda Hardie, Robert Palmer, Lynn Choo, David Hillman, Michael Hensley, Erin Kelty, Kevin Murray, Bhajan Singh, Bradley Wibrow

**Affiliations:** 1 Intensive Care Unit, Department of Anaesthesia, Intensive Care and Pain Medicine, John Hunter Hospital, Lookout Road, New Lambton Heights, NSW 2305 Australia; 2 Sir Charles Gairdner Hospital, Hospital Avenue, Nedlands, Perth, WA 6009 Australia; 3 Curtin University, Kent Street, Bentley, WA 6102 Australia; 4 University of Western Australia, 35 Stirling Highway, Crawley, WA 6009 Australia; 5 Department of Psychiatry, Royal Perth Hospital, GPO Box X2213, Perth, WA 6847 Australia; 6 Sir Charles Gairdner Hospital, Perth, WA Australia; 7 John Hunter Hospital, Lookout Road, New Lambton Heights, NSW 2305 Australia; 8 Sleep Department, Sir Charles Gairdner Hospital, Hospital Avenue, Nedlands, WA 6009 Australia; 9 School of Medicine, University of Western Australia, 35 Stirling Highway, Crawley, Perth, WA 6009 Australia; 10 Respiratory and Sleep Medicine Department, John Hunter Hospital, Lookout Road, New Lambton, NSW 2305 Australia; 11 School of Population Health, University of Western Australia, 35 Stirling Highway, Crawley, Perth, WA 6009 Australia; 12 Centre for Applied Statistics, University of Western Australia, 35 Stirling Highway, Crawley, Perth, WA 6009 Australia; 13 Pulmonary Physiology and Sleep Medicine, Sir Charles Gairdner Hospital, Hospital Avenue, Nedlands, Perth, 6009 WA Australia; 14 West Australian Sleep Disorders Research Institute, Internal Mailbox 201, , Queen Elizabeth II Medical Centre, Hospital Avenue, Nedlands, Perth, WA 6009 Australia; 15 Faculty of Science, Sir Charles Gairdner Hospital, Hospital Avenue, Nedlands, Perth, WA 6009 Australia; 16 Sir Charles Gairdner Hospital, Hospital Avenue, Nedlands, Perth, WA 6009 Australia; 17 University of Western Australia, 35 Stirling Highway, Crawley, Perth, WA 6009 Australia

**Keywords:** Delirium, Melatonin, Intensive care unit, Sleep

## Abstract

**Background:**

Delirium is an acute state of brain dysfunction characterised by fluctuating inattention and cognitive disturbances, usually due to illness. It occurs commonly in the intensive care unit (ICU), and it is associated with greater morbidity and mortality. It is likely that disturbances of sleep and of the day-night cycle play a significant role. Melatonin is a naturally occurring, safe and cheap hormone that can be administered to improve sleep. The main aim of this trial will be to determine whether prophylactic melatonin administered to critically ill adults, when compared with placebo, decreases the rate of delirium.

**Methods:**

This trial will be a multi-centre, randomised, placebo-controlled study conducted in closed ICUs in Australia. Our aim is to enrol 850 adult patients with an expected ICU length of stay (LOS) of 72 h or more. Eligible patients for whom there is consent will be randomised to receive melatonin 4 mg enterally or placebo in a 1:1 ratio according to a computer-generated randomisation list, stratified by site. The study drug will be indistinguishable from placebo. Patients, doctors, nurses, investigators and statisticians will be blinded. Melatonin or placebo will be administered once per day at 21:00 until ICU discharge or 14 days after enrolment, whichever occurs first. Trained staff will assess patients twice daily to determine the presence or absence of delirium using the Confusion Assessment Method for the ICU score. Data will also be collected on demographics, the overall prevalence of delirium, duration and severity of delirium, sleep quality, participation in physiotherapy sessions, ICU and hospital LOS, morbidity and mortality, and healthcare costs. A subgroup of 100 patients will undergo polysomnographic testing to further evaluate the quality of sleep.

**Discussion:**

Delirium is a significant issue in ICU because of its frequency and associated poorer outcomes. This trial will be the largest evaluation of melatonin as a prophylactic agent to prevent delirium in the critically ill population. This study will also provide one of the largest series of polysomnographic testing done in ICU.

**Trial registration:**

Australian New Zealand Clinical Trial Registry (ANZCTR) number: ACTRN12616000436471. Registered on 20 December 2015.

**Electronic supplementary material:**

The online version of this article (doi:10.1186/s13063-016-1751-0) contains supplementary material, which is available to authorized users.

## Background

Delirium is a state of acute organic brain dysfunction with fluctuating disturbances of attention and cognition which occurs as a direct consequence of an underlying medical condition [1]. It is particularly common in patients in intensive care units (ICUs), with a wide range of prevalence ranging between 11% and 83% reported in the literature [2–5]. Researchers in a multi-centre study that included one of the participating hospitals of this trial reported a delirium prevalence of 45% [5], whereas a preliminary audit for this trial at the two primary sites demonstrated a delirium prevalence of 52% when evaluating patients with an expected ICU length of stay (LOS) of more than 24 h.

Delirium in intensive care is associated with worse outcomes. These include higher mortality (estimated as a 10% increase in the relative risk of death for each day of delirium) [6–10], more time on a ventilator [9–11], unplanned removal of tubes and catheters [10], increased use of physical restraints [11], increased requirement for tracheostomy, greater post-operative complications [12], poorer functional status, cognitive impairment [7, 9, 13, 14], increased hospital and ICU LOSs [8, 11, 12], increased medical costs [15], higher risk of dementia [7, 13] and greater likelihood of admission to residential care [7].

The aetiology of delirium is multi-factorial [16]. In the critical care setting, it is likely that disturbances of sleep and of the normal circadian rhythm play a significant role [17]. Sleep deprivation may occur as a result of environmental disturbances, loss of normal day-night routine, critical illness itself and patient treatments. However, although sleep disturbances have been demonstrated to predispose individuals to post-operative delirium, sleep problems and their relationship to delirium in ICU remain relatively unexplored [18, 19].

In the clinical setting delirium can be approached with prevention strategies or treatment strategies, with the former appearing to be more effective. Authors of a recent meta-analysis [20] reported 8 of 15 preventative studies were successful, compared with 1 of 10 treatment studies, in terms of reducing delirium incidence and duration. There are several non-pharmacological strategies used for preventing delirium in ICU, but there are no pharmacological strategies that are used routinely [6, 20]. Reasons for this include a lack of clear evidence, limited pathophysiological understanding, potential side effects, cost and difficulties identifying which patients will benefit [21, 22].

Good-quality sleep at night is rapidly being recognised as an important aspect of delirium prevention [19]. Melatonin is a natural hormone secreted by the pineal gland, which helps in the regulation of the sleep-wake cycle [23]. It is stimulated by low light, peaking during the main sleep period [24–26]. Melatonin can be given exogenously. In healthy volunteers, it decreases sleep onset latency, increases sleep efficiency and increases total sleep duration [27]. It also increases the duration of rapid eye movement (REM) sleep, which is thought to have a positive effect on health and healing [28]. It is inexpensive, has an excellent safety profile and is widely used in the community [29–31]. Critical illness causes deregulation of melatonin secretion by the pineal gland, and light and darkness fail to restore its normal pattern of secretion [32, 33]. Serum melatonin levels are found to be lower after surgery, in delirious post-surgical patients and in those receiving opioids [25, 34]. Studies have also demonstrated that exogenous melatonin is well absorbed enterally in critically ill patients [35].

Melatonin has previously been evaluated for the treatment of delirium, but only five trials have put it to the test as a prophylactic agent. Sultan and colleagues [36] evaluated prophylactic melatonin in patients requiring elective hip replacement surgery and found a significant decrease in delirium in the group receiving melatonin. Al-Aama and colleagues [37] compared prophylactic melatonin with placebo in a population of acutely ill elderly patients and found that melatonin was associated with a reduction in the incidence of delirium. De Jonghe and colleagues [38, 39] evaluated the effect of prophylactic melatonin versus placebo on the incidence of delirium in patients requiring hip surgery and found no significant difference. Hatta and colleagues [40] compared the melatonin receptor agonist ramelteon prophylactically with placebo in critically ill medical patients and found a significant reduction in delirium in the ramelteon group. Artemiou and colleagues [41] evaluated the use of prophylactic melatonin in patients undergoing cardiac surgery. They reported a delirium incidence of 8.4% versus 250 control patients in whom the incidence was 20.8%. However, there was no placebo in their trial, and the trial was not blinded.

### Rationale for proposed study

Delirium is common in ICU; it is associated with worse outcomes when it occurs; and currently there are limited therapies to prevent it. The studies evaluating melatonin as a prophylactic agent suggest that melatonin could play an important role in preventing delirium in the critically ill [19]. However, these studies were small and conducted with populations that do not include ICU patients. It is unclear whether these proposed benefits would be replicated in an adequately powered, randomised controlled trial or if they would translate to critical care. In the present study, we will attempt to answer the question whether, in a population of critically ill adults, prophylactic melatonin, compared with placebo [42], decreases the prevalence, duration and severity of delirium.

### Objectives

The primary objective of this trial will be to determine whether melatonin given prophylactically decreases the prevalence of delirium in critically ill patients. The secondary objectives will be to determine whether prophylactic melatonin decreases the severity and duration of delirium, improves sleep quality, reduces hospital and ICU LOS, improves morbidity and mortality, and reduces healthcare costs through reduced LOS and intensity of medical therapy.

### Trial design

This study will be a prospective, multi-centre, randomised, placebo-controlled, double-blind, prophylactic intervention trial of melatonin 4 mg versus placebo given at 21:00 for 14 days or until ICU discharge to critically ill patients admitted to ICU with an expected ICU LOS of at least 72 h (two arms, 1:1 allocation, parallel design). A subgroup of 100 patients from the two main research sites (Sir Charles Gairdner Hospital and John Hunter Hospital) will also undergo polysomnographic testing.

## Methods

### Participants and settings

This study will be conducted in closed ICUs of participating hospitals in Australia. ICUs currently involved are all in university-affiliated teaching hospitals. We will recruit 850 patients admitted to ICU with an expected LOS of at least 72 h.

### Eligibility criteria

#### Inclusion criteria

Patients admitted to a participating ICU identified by the treating intensivist as having an expected total ICU LOS of 72 h or more are eligible. Patients must be enrolled within 48 h of their ICU admission and receive the first dose of the study drug on the day of enrolment.

#### Exclusion criteria

The exclusion criteria are patients younger than 18 years of age; patients already receiving melatonin therapy before their admission to ICU; prior hypersensitivity reaction to any of the components (sucralose and glycerol) of the study drug; patients expected to be discharged within 72 h of admission; expected/inevitable death within 48 h of enrolment; pregnancy or breastfeeding; patients who are non-English-speaking; patients whose condition is not expected to improve adequately for them to be able to be assessed with a Confusion Assessment Method for the ICU (CAM-ICU) score during their ICU stay; patients who are not able to be assessed because of neurological problems that would affect their ability to participate in CAM-ICU assessment (as judged by the treating physician); no enteral route/nil by mouth (melatonin is not available in intravenous formulation); and hepatic impairment, defined as alanine aminotransferase greater than 500 IU/L, previous liver transplant or liver cirrhosis of Child-Pugh classes B and C.

### Interventions

Eligible participants will be randomised to receive melatonin 4 mg or placebo at 21:00 for 14 consecutive nights or until ICU discharge, whichever occurs first. This will be given either orally or via nasogastric tube, depending on the circumstances. All concomitant care and interventions that are normal practice for the ICU or based on other trials are permitted.

A subset of 100 patients will also undergo two episodes of polysomnographic testing overnight on days 2–3 and days 5–7 of their ICU stay. This will occur only at the two primary sites: Sir Charles Gairdner Hospital and John Hunter Hospital.

### Outcomes

All assessors using the CAM-ICU and the Confusion Assessment Method for delirium severity (CAM-S) will receive training in these assessment tools along with regular monitoring to ensure the tools are being applied reliably. The primary outcome will be prevalence of delirium. This will be measured and reported as delirium-free assessments. The secondary outcomes will be (1) overall prevalence of delirium, (2) duration of delirium, (3) severity of delirium, (4) sleep quality, (5) ICU and hospital LOS, (6) morbidity and mortality and (7) healthcare costs.

#### Prevalence of delirium

The primary outcome will be the proportion of delirium-free assessments as measured by the CAM-ICU score. Prevalence rather than incidence has been chosen because delirium on admission to ICU may not be measureable, owing to many patients being intubated and sedated and therefore unable to be assessed. Where possible, we will measure baseline delirium and provide an incidence in those patients. The CAM-ICU is widely used and is the best-performing of the tools adapted for use in ICU by non-psychiatrists, with a pooled sensitivity of 80.5% and specificity of 95.9% [43, 44]. The CAM-ICU will be used twice daily with all assessable patients while they are receiving the study drug for 14 days or until ICU discharge, whichever occurs sooner.

Secondary outcomes will include the following:

1. *Overall prevalence of delirium*: Patients will be diagnosed as delirious when they have at least one positive CAM-ICU up to ICU discharge or day 14 after enrolment if still in ICU.

2. *Duration of delirium*: Once the diagnosis of delirium is made, duration will be recorded as how many subsequent days on which they have a positive CAM-ICU until ICU discharge or day 14 after enrolment.

3. *Severity of delirium*: Severity will be assessed using the CAM-S Short Form [45], which gives a score from 0 to 8. Considering that sleep is an important part of this study, an additional question from the CAM-S Long Form on the sleep-wake cycle has been included in the assessment. Thus, a score from 0 to 10 will be recorded.

Indirect markers of delirium severity will also be recorded daily until ICU discharge or day 14 after enrolment. These are (1) need for anti-psychotics or sedation (total daily doses will be calculated); (2) need for physical restraints; (3) patient participation in physiotherapy sessions and mobilisation; and (4) patient removal of intravenous lines, drains or catheters.

#### Sleep quality

Sleep quality will be measured by (1) daily patient/nurse-rated sleep assessment (Richards-Campbell Sleep Questionnaire) while in ICU [46] and (2) patient-rated sleep quality questionnaire after ICU discharge [47]. This will be performed from 2 to 14 days after ICU discharge with the timing dependent on the patient’s ability to participate. Patients unable to participate will be omitted.

A subgroup of 100 patients will undergo sleep quality assessments twice during their ICU stay using polysomnography. The hypothesis is that melatonin will increase the quality and quantity of sleep compared with placebo. These 100 patients will come only from either Sir Charles Gairdner Hospital or John Hunter Hospital. Patients included in the study at these sites will be sequentially allocated to the subgroup. Polysomnography will be done between days 2 and 3 and between days 5 and 7 of the ICU stay. For logistical reasons, we will have limited access to polysomnography. It will be available only from Monday through Thursday, and hence we have not been able to randomise patients to this investigation. It is expected that, by virtue of blinding and randomisation, approximately 50% of patients will be from the melatonin arm and 50% from the placebo arm. The polysomnography protocol and reporting have been standardised and agreed upon by senior sleep physicians at the two main centres. Patients will not be in a single room every time polysomnography is performed, because this is a pragmatic study and will reflect real-world conditions as much as possible. Many participating units do not have single rooms for all of their patients. The range of days is selected so that if patients cannot undergo polysomnography because of procedures, they could have it the following night. If polysomnography needs to be aborted, we will try to repeat it within that range of days. This will aid in the understanding of sleep patterns in the critically ill and the biological mechanisms through which melatonin may work.

Several parameters will be generated by the sleep studies. Two sleep experts involved in the study design have selected the following four variables as the most important (each will be a secondary outcome): (1) total sleep time, (2) sleep efficiency, (3) percentage REM sleep and (4) arousal index.

#### ICU and hospital length of stay

ICU and hospital length of stay will be recorded using existing hospital patient data programmes linked to medical records.

#### Morbidity and mortality

Standard ICU markers of morbidity and mortality will be recorded on a daily basis until ICU discharge or day 14 after enrolment. These include (1) duration of mechanical ventilation measured as ventilator-free days; (2) daily need for vasopressor use; (3) daily need for renal replacement therapy; (4) re-intubation, delayed extubation or need for tracheostomy; (5) Sequential Organ Failure Assessment score for injury severity; (6) mortality at 28 and 90 days; and (7) destination after ICU discharge.

#### Healthcare costs

A medical economist will perform a cost-consequence analysis based on LOS and intensity of medical therapy while in ICU.

### Participant timeline and recruitment

**Fig. 1:**
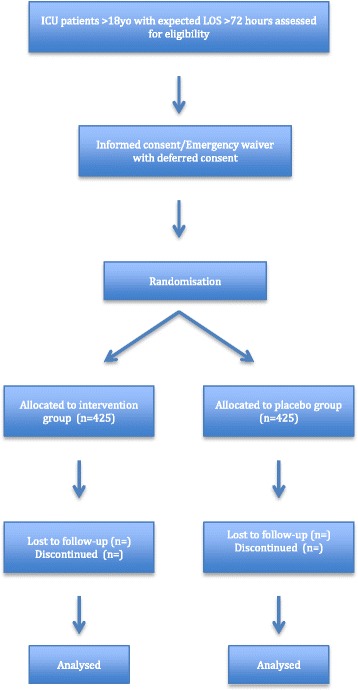
Time schedule of Prophylactic Melatonin for Delirium in Intensive Care (Pro-MEDIC) enrolment, interventions and assessments. *CAM-ICU* Confusion Assessment Method for the ICU, *CAM-S* Confusion Assessment Method for delirium severity, *ICU* Intensive care unit, *LOS* Length of stay

Patients will be screened on the morning handover for eligibility. Baseline data will be recorded on day 0. Patients will be assessed each day and have the primary and secondary outcomes recorded daily until ICU discharge or day 14 after enrolment. Patients will be seen on the ward between days 2 and 14 after ICU discharge to complete the sleep questionnaire. Patients who have been discharged home will be contacted by phone. Patients will be followed through their hospital stay to record ICU LOS, hospital LOS, and 28- and 90-day mortality. The trial assessment schedule is displayed in Table 1 and Fig. 1.

**Table 1:** Time schedule of Pro-MEDIC enrolment, interventions and assessments

	Day 1	Day 2	Day 3	Day 4	Days 5–14	Days 2–14 post-ICU discharge	Hospital discharge	Days 28 and 90
Inf`ormed consent	X							
Demographic information	X							
Medical co-morbidities	X							
Psychiatric history	X							
Liver function tests	X				X (days 0, 5 and 10)			
CAM-ICU score	X	X	X	X	X			
CAM-S test (for patients found to be delirious)	X	X	X	X	X			
Record daily ICU interventions	X	X	X	X	X			
Record daily anti-psychotic and sedation use	X	X	X	X	X			
Record daily sleep aid measures	X	X	X	X	X			
RCSQ	X	X	X	X	X			
Use of physical restraints	X	X	X	X	X			
Physiotherapy participation	X	X	X	X	X			
Polysomnography		X			X			
Assessment of sleep quality						X		
ICU and hospital LOS							X	
Hospital discharge destination							X	
Mortality at 28 and 90 days								X

*Abbreviations: CAM-ICU* Confusion Assessment Method for the ICU, *CAM-S* Confusion Assessment Method for delirium severity, *ICU* Intensive care unit, *LOS* Length of stay, *Pro-MEDIC* Prophylactic Melatonin for Delirium in Intensive Care, *RCSQ* Richards-Campbell Sleep Questionnaire

To allow for accurate recording of LOS, we will allow for a 6-month period after the last patient is enrolled. If there are patients still hospitalised at that point, the last day of the 6-month period will be recorded to calculate as their last day of hospital admission.

### Sample size

On the basis of two sample size calculations, with an alpha value of 0.05 and a power of 80%, a total of 850 patients will be recruited into the study, with 425 patients in each arm. Estimates of sample size were made on the basis of percentage of delirium-free assessments (the primary outcome). An audit of 100 patients at Sir Charles Gairdner Hospital and John Hunter Hospital was used to obtain preliminary data to guide sample size calculations where an average of 54% of assessments were deemed delirium-free (SD 45%). The research group is aiming to detect a 10% increase in the percentage of delirium-free assessments. This would give us a sample size calculation of 319 per group. Adjustment for a non-parametric test (15%) increases this to 367 per group. It is expected that up to 10% of patients recruited into the study will not be assessable for delirium at any point during the study. To account for this and for missing data/loss to follow-up, the sample size was increased by 15% to 423 patients per group (total rounded to 850). This sample size will also be sufficient to analyse the secondary measures.

To prevent and treat the issue of likely missing data, the follow-up period for the primary outcome is short and data collection is electronic, with out-of-range and missing alerts. There will be documentation of why data are missing. This will help us to formulate reasonable assumptions about observations that are missing [48]. To achieve adequate recruitment numbers, there will be frequent reminders to the participating sites, and progress reports will be scheduled periodically.

### Allocation, randomisation, compliance and blinding

Patients will be randomised to melatonin or placebo in a 1:1 ratio according to a computer-generated randomisation list, stratified by site, with standard block sizes of six. An independent statistician other than the statistician analysing the data will create the allocation sequence. This randomisation list will be provided by the independent statistician directly to the compounding pharmacy. Each patient’s bottle will be labelled with a site-specific randomisation number, which will become that patient’s study number. Study sites will administer the content of the study drug bottles sequentially to patients in the order in which they are included in the study.

Each participating site’s principal investigator or research coordinator will enrol patients. All patients will receive the same interventions, except for the sleep studies, which will be assigned by the principal investigators at the two sites performing sleep studies. A flowchart of enrolment, allocation, follow-up and analysis is shown in Additional file 1.

When a patient is enrolled in the study, ‘Pro-MEDIC study drug’ will be charted on the medication chart. The ICU bedside nurse, using an oral/enteral syringe, will administer the study drug at 21:00 until discharge from ICU or 14 days after enrolment, whichever occurs first.

The study drug will be prepared by a single private compounding pharmacy that currently produces melatonin suspension for the health department and has extensive experience in clinical trials. They will be given the randomisation list and will prepare individual bottles for each patient that will contain an amount of the study drug to last for up to 14 days. Both melatonin and placebo will be made to look and taste the same. The melatonin will be prepared as a 2 mg/ml solution. The drug packs will be shipped from the compounding pharmacy to each of the participating sites, and responsibility will be assigned to the site’s principal investigator.

The shelf life of the melatonin suspension is 12 months. The project manager will co-ordinate with the pharmacy to ensure sites have an adequate supply of the study drug based on recruitment rates. Subjects may withdraw their consent to participate in the study at any time and for any reason.

Compliance will be indicated by the nurse’s signature on the medication chart each day that the study drug is administered to the patient. There will be no restriction or change in the use of any sedative, analgesic or anti-psychotic agents given, but the use of these medications will be recorded.

The patients, the investigators, and the ICU doctors and nurses looking after patients enrolled in the study will be blinded to the treatment that patients are receiving. Only the independent statistician providing the allocation list to the pharmacy, the compounding pharmacy, and the independent data and safety monitoring committee (DSMC), should they need to investigate an adverse event (AE), will know which is melatonin and which is placebo.

A standardised process for unblinding will be in place for all sites. Blinding will be broken only if the senior treating ICU doctor feels that the drug is causing AEs to the patient and it is necessary to know if the patient received melatonin, such as in the case of anaphylaxis. Other AEs may lead to cessation of the study drug but would not be expected to require unblinding.

### Data collection and management

Data will be collected and entered into a REDCap© [49] electronic database behind a secure firewall. The REDCap system will perform data entry validation. For the non-primary sites, data can be entered into the REDCap database, or the case report forms can be filled in manually and forwarded to the primary sites. Strict confidentiality and privacy will be kept at all times. The data will be stored in password-protected files and will be kept for 15 years. The final database will be locked after all information for the last patient has been entered and provided to the statistician.

If a participant withdraws consent, the investigators will ask permission to retain all data collected up to the point of withdrawal and to collect survival status and discharge destination data. If subjects cease the study drug for any reason, data will still be collected and interpreted in an intention-to-treat analysis. The reason for withdrawal will be recorded. No attempt will be made to replace patients who withdraw from the study.

### Statistical methods

Analyses will be conducted on both intention-to-treat and per-protocol bases. All outcomes will be analysed for superiority (two-sided) and considered statistically significant at the 5% level. Baseline data will be recorded and analysed for imbalances between the treatment groups. The primary endpoint, percentage of delirium-free assessments, will initially be analysed using a Mann-Whitney *U* test to compare the two treatment arms. Subsequent supporting analyses will be carried out using a negative binomial model with adjustments made for number of assessments (accounting for withdrawals), important covariates and baseline data where appropriate. Secondary endpoints of any prevalence of delirium during the 14 days and duration of delirium from first onset will be analysed using a chi-square test with supporting analyses of binary and ordinal logistic regression, respectively, allowing adjustments for covariates. Sleep quality analysis based on the Richards-Campbell Sleep Questionnaire on each of days 2–14, the patient-rated sleep questionnaire completed after day 14, and sleep measurements derived from polysomnography done on days 2–4 and 5–7 (total sleep time, sleep efficiency, REM sleep percentage and arousal index) will all be analysed initially using a Mann-Whitney *U* test to compare the two treatment arms and subsequently using linear mixed models with fixed factors of treatment time and their corresponding interaction, along with random patient effects. ICU LOS, total hospital LOS, mortality and morbidity events will be analysed initially using Kaplan-Meier curves and log-rank tests and subsequently using Cox proportional hazards models. Individuals withdrawn from the study early will be censored at the date of withdrawal. Specific subgroup analysis of major outcomes will be carried out, stratifying by age, sex, delirium medical subgroup and delirium risk as per the prediction of delirium in ICU patients (‘PRE-DELIRIC’) score. A fully specified statistical analysis plan will be finalised and approved by statisticians at the two main hospitals before the database is unlocked and opened. Data analysis is to be carried out using the R software programme [50].

### Data monitoring and safety

A DSMC will be made up of an independent intensivist, an epidemiologist and a statistician. They will confer after 100 patients have been enrolled and will review the unblinded data to determine the need for early termination of the trial. Early termination will be decided on the basis of either (1) safety concerns with the ongoing review of all severe adverse events (SAEs) and suspected unexpected severe adverse reactions (SUSAR) or (2) outstanding benefits. The DSMC will report to the research committee and make a recommendation after its review. A similar interim analysis will be performed at the midpoint of the study after enrolment of 400 patients.

The Haybittle-Peto stopping rule will be used in both analyses. Thus, if there is a probability of less than 0.001 in either direction that the treatment and placebo are different, the DSMC will recommend that the study be ceased.

Melatonin has no serious adverse reactions documented in the medical literature [51]. An AE caused by either melatonin or its solution will be considered as any untoward medical occurrence in a patient enrolled in this study, regardless of its causal relationship to the study treatment. AEs will be monitored with a checklist of symptoms commonly associated with medications, including headache, gastrointestinal symptoms, lethargy, myalgias and skin symptoms.

Melatonin has a very safe therapeutic profile. Assessment and documentation of AEs will be carried out by the principal and associate investigators and will be included in the data collection instrument. Once collected, these will be reviewed and classified as AEs, SAEs or SUSARs. The DSMC will have access to this information as unblinded data.

The baseline mortality of ICU patients enrolled in trials will be high because of the critical illness necessitating admission. New organ failure in a high proportion of ICU patients is not unexpected, regardless of optimal treatment and study intervention. As such, and in keeping with the advice of Cook and colleagues, events that are part of the natural history of the primary disease process or expected complications of critical illness will not be reported as SAEs in this trial unless they are thought to be causally related to the study intervention [52].

### Ethics and dissemination

Ethics approval has been sought and obtained from the Sir Charles Gairdner Hospital Human Research Ethics Committee in Western Australia and the Hunter New England Human Research Ethics Committee in New South Wales. The NSW Civil and Administrative Tribunal (NCAT) has granted approval to conduct the clinical trial in this state. Protocol amendments will be communicated to these ethics committees for approval.

Subject confidentiality is strictly held in trust by the investigators and is extended to clinical data collected and test results. No information will be released to any third party without the prior written approval of the participating institutions. Clinical data will not be released without the written permission of the participating individuals, except for the DSMC where required. Data will be identified only by the study’s subject identification number.

This study protocol has been written following the Standard Protocol Items: Recommendations for Interventional Trials (SPIRIT) checklist (Additional file 1). A populated checklist (Table 1) and a figure (Additional file 1) displaying the schedule of enrolment, interventions and assessments have been provided. The results of this trial will be written as an article that will be submitted to peer-reviewed medical journals for publication.

### Consent

Informed consent will be obtained from the patients or, when it is not possible for patients to consent for themselves, from their person responsible. The investigators received approval from the NCAT to allow the inclusion of patients who are highly dependent on medical care. In Western Australia, the study will be undertaken using an emergency waiver of consent, as allowed for in the National Health and Medical Research Council national statement, in conjunction with acknowledgement from the patient’s person responsible that he or she is not aware of any reason why the patient would have chosen not to participate. All patients will be followed and given the opportunity to consent to continue in the study once their capacity to consent is regained [53].

## Discussion

Delirium is increasingly being recognised as a significant issue in hospital and particularly in ICU. It is associated with poorer outcomes for the patients, their families and the wider community. Intensive care is an area where the prevalence of delirium is higher than in the general wards. There are multiple potentially modifiable risk factors but very limited effective pharmacological options for either prevention or treatment.

To our knowledge, this trial will be the largest trial evaluating melatonin as a prophylactic agent for delirium in any setting, and certainly the largest in intensive care. It will also be one of the larger trials evaluating any prophylactic agent in intensive care. Currently, a trial by van den Boogaard and colleagues is recruiting. They are evaluating haloperidol as a preventative agent for ICU delirium and plan to enrol 2145 patients into 3 arms. However, haloperidol has potential side effects, whereas melatonin is extremely safe, inexpensive and potentially more acceptable to patients, with the rate of side effects being less than placebo [51].

The polysomnography arm will be an insightful explanatory component of the study and, to our knowledge, will be one of the largest series of polysomnography in the ICU setting. It may open up further avenues for research into sleep in critical care.

The choice of 4 mg of melatonin as a dose required some consideration. The existing trials have used a variety of doses ranging from 0.5 mg to 5 mg [37–39, 41]. Bourne and colleagues [54] used 10 mg to aid sleep and found a carry-over effect to the following day. Therefore, we feel that 4 mg is a practical dose within the range of previous studies. Evidence exists confirming absorption in critical care for similar doses, and this dose is unlikely to have a significant carry-over effect the following day. Melatonin (Circadin; Flynn Pharma, Dublin, Ireland), the current Pharmaceutical Benefits Scheme listed agent, also comes in 2-mg tablets.

Patients will be assessed for delirium on enrolment. This will not be possible where patients arrive at the unit ventilated and heavily sedated. Because of this, true incidence will not be able to be established, and it is recognised that some patients may already have delirium on enrolment. The investigators feel it is important to keep this a pragmatic trial. The decision to mandate that patients be enrolled within 48 h of their ICU admission was made to ensure that the study drug is given early in patients’ stay.

The enrolment of patients who have an expected LOS longer than 72 h is being done because patients with increased LOS are more likely to develop delirium. Although prediction tools for delirium do exist [55, 56], they are complex, and we seek to maintain the generalisability of our findings to a broad range of ICU patients.

### Trial status

We are in the process of recruiting patients.

## Additional file

Additional file 1: SPIRIT 2013 checklist: recommended items to address in a clinical trial protocol and related documents. (DOC 121 kb)

## Abbreviations

AE: Adverse event
CAM-ICU: Confusion Assessment Method for the ICU
CAM-S: Confusion Assessment Method for delirium severity
CRF: Case report form
D/C: Discharge
DSMC: Data and safety monitoring committee
ICU: Intensive care unit
LOS: Length of stay
NCAT: NSW Civil and Administrative Tribunal
PRE-DELIRIC: Prediction of delirium in ICU patients
Pro-MEDIC: Prophylactic Melatonin for Delirium in Intensive Care
RCSQ: Richards-Campbell Sleep Questionnaire
REM: Rapid eye movement
SAE: Severe adverse event
SPIRIT: Standard Protocol Items: Recommendations for Interventional Trials
SUSAR: Suspected unexpected severe adverse reaction
